# Biomechanical evaluation of diabetic foot through hierarchical cluster analysis

**DOI:** 10.1186/1757-1146-7-S1-A72

**Published:** 2014-04-08

**Authors:** Zimi Sawacha, Fabiola Spolaor, Gabriella Guarneri, Annamaria Guiotto, Angelo Avogaro, Claudio Cobelli

**Affiliations:** 1 Department of Information Engineering, University of Padova, Italy; 2 Department of Clinical Medicine and Metabolic Disease, University Polyclinic, Padova, Italy

## Introduction

Type 2 diabetes is predicted to become the 7^th^ leading cause of death in the world by the year 2030 [1]. Diabetic foot is the most common long-term diabetic complication, and it is a major risk factor for plantar ulceration (PU), it is determined by peripheral neuropathy (PN), vascular disease, increased foot pressures, foot trauma, deformity and callus [1].

The aim of this study is to develop a methodology for automatic detection of patients at risk for PU based on 3 dimensional (3D) multisegment foot biomechanics through cluster analysis.

## Methods

For this purpose 44 subjects, 20 with (PN) and 24 without PN (noPN) were enrolled. Simultaneous kinematic, kinetic and plantar pressure (PP) data were acquired during gait with a BTS motion capture system (6 cameras, 60-120 Hz) synchronized with 2 Bertec force plate (FP4060-10) and 2 Winpod pressure plate as in [2]. After gait analysis 5 years clinical follow up was performed on each subject including: neuropathy diagnosis following ADA recommendation as in [2,3], electroneurophysiological study; Index of Winsor, cardiovascular autonomic tests, HbA1c values, micro-macroalbuminuria values, a carotid artery Doppler ultrasound examination.

A hierarchical cluster (HC) technique was adopted [3] using TimeClust1.1. In the present work kinematics, kinetics and PP data estimated as in [2] were used as input. Peak value and its position in term of stance phase of gait’s percentage was extract from each variable. HC was performed either using each type of variable and putting them all together as input or by using each type of variable separately (3D kinematics, kinetics, PP). In order to explore how the subjects were distributed in the proposed cluster, descriptive statistics was used. Statistical differences of both biomechanics and clinical variables between the obtained clusters were investigated using Student T-test and Pearson correlation (MatlabR2011b). After 5 years follow up 3 subjects ulcerated.

## Results

**Figure 1:**
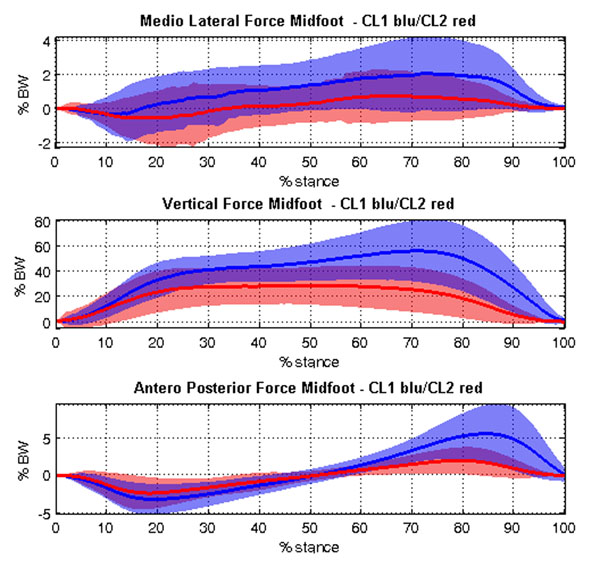
Peak of Medio Lateral, Vertical and Anterior Posterior Force of Midfoot in each cluster (in blu Cluster 1, in red Cluster 2).

Results of HC analysis (see Table 1 and Figure 1) performed either using only 3D subsegments kinematics or kinetics defined two groups, one including PU subjects and one not. The cluster containing PU subjects was characterized by larger number of diabetes complications and higher values of biomechanics variables.

**Table 1:** Clinical data of subjects for each cluster. In the upper part of table are collected data of cluster using Ground Reaction Force (GRF) input; in the lower part are collected data of luster using kinematics input.

GRF	CL 1		CL 2		p Value
**Subjects per Cluster**	11		32		

	**Mean and St. Dev**				

**Year of Disease**	16,73	10,80	20,68	12,61	

**HbA1c**	7,97	1,26	7,96	1,14	

	**Presence of Complications**				

**Vasculopathy**	3 (27.27%)		5 (15.625%)		

**microalbuminuria**	2 (18.18%)		4 (12.5%)		

**Neuropathy**	3 (27.27%)		17 (53.125%)		

**Autonomic Neuropathy**	2 (18.18%)		7 (21.875%)		

**Finger Deformity**	1 (9.09%)		13 (40.625%)		

**Callosity**	2 (18.18%)		18 (56.25%)		0,02926

**Ulcer**	0		3(9.37%)		

**KINEMATICS**	**CL 1**		**CL 2**		**p Value**

**Subjects per Cluster**	18		26		

	**Mean and St. Dev**				

**Year of Disease**	23,67	11,82	16,44	11,56	

**HbA1c**	7,95	1,27	7,97	1,11	

	**Presence of Complications**				

**Vasculopathy**	3 (16.67%)		5 (19.23%)		

**microalbuminuria**	3 (16.67%)		3 (11.54%)		

**Neuropathy**	6 (33.33%)		8 (30.77%)		

**Autonomic Neuropathy**	10 (55.56%)		10 (38.46%)		

**Finger Deformity**	12 (66.67%)		9 (34.62%)		0,03662

**Callosity**	5 (27.78%)		4 (15.39%)		

**Ulcer**	0		3(11.5%)		

## Conclusions

In conclusion, our work highlighted the presence of warning signs of neuropathy even in diabetic subjects without a clinical diagnosis of PN. Furthermore 2 type of variables were able to correctly identify the 3 subjects who developed PU within the 5 years (e.g. 3D foot kinematics and kinetics).

## References

[B1] American Diabetes Association American Academy of Neurology Consensus Statement. Report and Recommendations of the San Antonio Conference on Diabetic Neuropathy Diabetes Care 1988 11 592 597 3060328 10.2337/diacare.11.7.592

[B2] Sawacha Z Guarneri G Cristoferi G Guiotto A Avogaro A Cobelli C Integrated kinematics–kinetics–plantar pressure data analysis: A useful tool for characterizing diabetic foot biomechanics Gait & Posture 2012 36 20 26 10.1016/j.gaitpost.2011.12.007 22464271

[B3] Magni P Ferrazzi F Sacchi L Bellazzi R TimeClust: a clustering tool for gene expression time series Bioinformatics 2008 24 3 430 2 10.1093/bioinformatics/btm605 18065427

